# Central role of IP_3_R2-mediated Ca^2+^ oscillation in self-renewal of liver cancer stem cells elucidated by high-signal ER sensor

**DOI:** 10.1038/s41419-019-1613-2

**Published:** 2019-05-21

**Authors:** Cuiwei Sun, Bo Shui, Wei Zhao, Hui Liu, Wenwen Li, Jane C. Lee, Robert Doran, Frank K. Lee, Tao Sun, Qing Sunny Shen, Xianhua Wang, Shaun Reining, Michael I. Kotlikoff, Zhiqian Zhang, Heping Cheng

**Affiliations:** 1 State Key Laboratory of Membrane Biology, Beijing Key Laboratory of Cardiometabolic Molecular Medicine, Peking-Tsinghua Center for Life Sciences, Institute of Molecular Medicine, Peking University, Beijing, 100871 China; 2 Department of Biomedical Sciences, College of Veterinary Medicine, Cornell University, Ithaca, NY 14853 USA; 3 Department of Cell Biology, Key Laboratory of Carcinogenesis and Translational Research (Ministry of Education/Beijing), Peking University Cancer Hospital and Institute, Beijing, 100142 China; 4 Third Department of Hepatic Surgery, Eastern Hepatobiliary Surgery Hospital, Second Military Medical University, Shanghai, 200438 China; 5 Beijing Key Laboratory of Cardiometabolic Molecular Medicine, Institute of Molecular Medicine, Peking University, Beijing, 100871 China

**Keywords:** Cancer stem cells, Mechanisms of disease

## Abstract

Ca^2+^ oscillation is a system-level property of the cellular Ca^2+^-handling machinery and encodes diverse physiological and pathological signals. The present study tests the hypothesis that Ca^2+^ oscillations play a vital role in maintaining the stemness of liver cancer stem cells (CSCs), which are postulated to be responsible for cancer initiation and progression. We found that niche factor-stimulated Ca^2+^ oscillation is a signature feature of CSC-enriched Hep-12 cells and purified α2δ1^+^ CSC fractions from hepatocellular carcinoma cell lines. In Hep-12 cells, the Ca^2+^ oscillation frequency positively correlated with the self-renewal potential. Using a newly developed high signal, endoplasmic reticulum (ER) localized Ca^2+^ sensor GCaMP-ER2, we demonstrated CSC-distinctive oscillatory ER Ca^2+^ release controlled by the type 2 inositol 1,4,5-trisphosphate receptor (IP_3_R2). Knockdown of IP_3_R2 severely suppressed the self-renewal capacity of liver CSCs. We propose that targeting the IP_3_R2-mediated Ca^2+^ oscillation in CSCs might afford a novel, physiologically inspired anti-tumor strategy for liver cancer.

## Introduction

Ca^2+^ signaling plays essential roles in the initiation and progression of diverse diseases including cancer. Recent studies have shown that dysregulation of Ca^2+^ homeostasis emerges as an important hallmark of tumor cells because remodeling of Ca^2+^ channels and transporters is common to tumor progression^1,2^ and Ca^2+^ oscillation orchestrates invadopodium formation and invasion during cancer metastasis^3^.

Cancer stem cells (CSCs) are thought to be the origin of cancer initiation and progression. It has been reported that Ca^2+^/calmodulin-dependent protein kinase IIγ prompts the stem-like properties of lung cancer cells^4^, while calcineurin, a Ca^2+^/calmodulin-dependent protein phosphatase, represses keratinocyte CSC potential^5^. Recently, we have shown that spontaneous Ca^2+^ oscillation occurs in the CSC-enriched liver cell line Hep-12^6^, and modulates the efficiency of self-renewal^6,7^. Ca^2+^ oscillation decodes the stimulations not only by its amplitude, but also by its frequency and duration, thereby expanding the capabilities of the signaling pathway^8–10^.

The endoplasmic reticulum (ER), which extends over the entire cytoplasm as an elaborated nanotubular network, acts as the most important intracellular Ca^2+^ store. However, investigation of ER Ca^2+^ signaling has been limited by a lack of ER-localized, high-signal sensors that can report store Ca^2+^ dynamics in real time. Circularly permutated EGFP variants fused with calmodulin and M13 motif, termed GCaMPs or G-GECOs are robust Ca^2+^ sensors that have undergone progressive improvements^11–15^, and for which the structural basis of Ca^2+^-dependent fluorescence has been determined^16^. While GCaMPs have been optimized for the detection of rapid cytosolic Ca^2+^ signaling, there has been limited success in creating variants with high dynamic range within the micromolar Ca^2+^ endoplasmic/sarcoplasmic environment. Recent progress has been made in this area using new classes of fluorescent protein sensors^17,18^, or GCaMP variants with mutated calmodulin moieties^18,19^. However, the brightness and dynamic range of these proteins, have been limited.

In the present study, we aimed to test the hypothesis that Ca^2+^ oscillation might play a vital role in maintaining the stemness of CSCs. We investigated the Ca^2+^ phenotypes, their subcellular and molecular mechanisms, their significance in cancer biology and potential strategies for intervening CSCs through manipulating Ca^2+^ signaling in CSC-enriched Hep-12 cells. In addition, we developed GCaMP-ER2, a novel ER localized Ca^2+^ sensor, to probe the store Ca^2+^ dynamics simultaneously. Our comprehensive approaches allow us to propose that targeting the IP_3_R2-mediated Ca^2+^ oscillation in CSCs might afford a novel, physiologically inspired anti-tumor strategy for liver cancer.

## Results

### Ca^2+^ oscillation is a signature feature of liver CSCs

To test the hypothesis that Ca^2+^ oscillation is a hallmark of CSCs, we applied a panel of potential niche factors, including ATP, epidermal growth factor (EGF)/basic fibroblast growth factor (FGFb)/B27, and interleukin (IL)-6, and assessed the Ca^2+^ response in the liver CSC line Hep-12 and its matching hepatocellular cancer (HCC) line Hep-11. ATP is frequently released from cells to facilitate metastasis^20^. EGF is a well-accepted niche factor and EGF/FGFb/B27 are commonly used in media for in vitro spheroid formation assays, the most popular end-point experiment for self-renewal determination in solid tumors^21,22^. IL-6 has been reported to elevate or maintain the self-renewal of liver CSCs^23^. Besides these, we opted to include methacholine, a muscarinic receptor agonist capable of inducing dynamic Ca^2+^ changes in many types of cells^24^. All of these stimulants are linked to the inositol 1,4,5-trisphosphate (IP_3_) signaling pathway and thereby trigger intracellular Ca^2+^ release from the ER via IP_3_ receptors (IP_3_Rs)^24–27^.

**Fig. 1:**
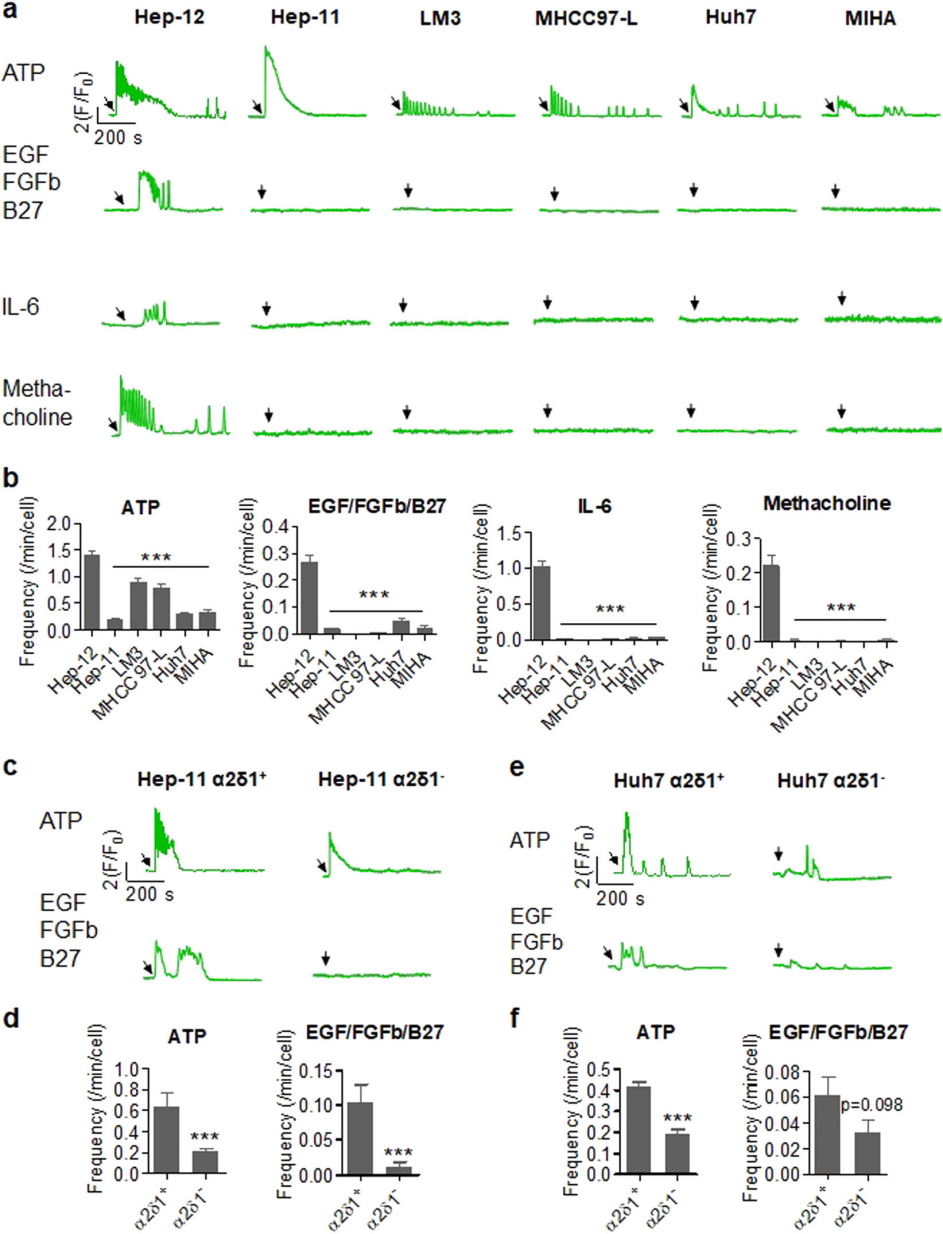
Ca^2+^ oscillation in liver cell lines responding to diverse stimuli. **a** Cytosolic Ca^2+^ dynamics in different cell types. The liver cancer stem cell line Hep-12 was compared to another liver cancer cell line derived from the same patient (Hep-11), other cancer cell lines (LM3, MHCC97-L, and Huh7), and a nontumorigenic immortalized liver cell line (MIHA). Representative time courses are shown as the normalized Fluo-4 fluorescence signal (*F*/*F*_0_, where *F*_0_ refers to basal fluorescence). Arrows mark the onset of stimulation. **b** Statistics of Ca^2+^ oscillation frequencies. The number of Ca^2+^ oscillations on top of the large, slow Ca^2+^ transient was counted over a 15-min window starting at the onset of stimulation (*n* *>* 100 cells/group. ***P *<* 0.001 versus Hep-12 cells). **c**–**f** Differential Ca^2+^ responses to ATP or EGF/FGFb/B27 stimulation in α2δ1^+^ and α2δ1^−^ cells from Hep-11 or Huh7 cells. Arrows in representative traces **c, e** mark the onset of stimulation. For statistics **d**, **f**, Hep-11: *n* *=* 40 (α2δ1^+^) and 86 cells (α2δ1^−^) for ATP stimulation; *n* = 36 (α2δ1^+^) and 69 cells (α2δ1^−^) for EGF/FGFb/B27 stimulation. Huh7: *n* *=* 120 (α2δ1^+^) and 137 cells (α2δ1^−^) for ATP stimulation; *n* = 109 (α2δ1^+^) and 94 (α2δ1^−^) cells for EGF/FGFb/B27 stimulation (***P *<* 0.001 versus α2δ1^+^ cells). All data were acquired with three independent experiments

We found that these stimulants each induced a large, slow Ca^2+^ transient that lasted several minutes in Hep-12 cells. Remarkably, Ca^2+^ oscillations of spiky appearance overlaid the evoked Ca^2+^ transient, and often continued even in the tail of the transient, when the cytosolic Ca^2+^ level returned to baseline (Fig. 1a, Movie S1). In contrast, typical Hep-11 cells did not show any Ca^2+^ change in response to most of the stimulants (EGF/FGFb/B27, IL-6, and methacholine), or exhibited only a smooth, monophasic Ca^2+^ transient in response to ATP (Fig. 1a, b, Fig. S1b, 2). That is, CSCs and HCCs exhibited distinct Ca^2+^ responses upon stimulation by self-renewal-related factors or IP_3_ pathway-related agonists.

We next extended the experiments to include multiple HCC lines, such as highly-metastatic LM3, poorly metastatic MHCC97-L, and Huh7 cells. ATP did elicit oscillatory Ca^2+^ responses in these cells, but their frequencies were significantly lower than those in Hep-12 cells (Fig. 1a, b). Further, none of the other three stimulants induced any oscillatory Ca^2+^ response in the vast majority of cells of each type (Fig. 1a, b). Similar results were found in the nontumorigenic immortalized liver cell line MIHA (Fig. 1a, b, Fig. S1b).

We have previously shown that α2δ1, a subunit associated with L-, P-, N-, and R-type Ca^2+^ channels, is a surface marker in liver CSCs^6,7^. We used the α2δ1 biomarker for sorting CSCs. More α2δ1^+^ Hep-11 cells than α2δ1^−^ cells displayed Ca^2+^ oscillation and the ensemble-averaged oscillation frequency was 3.1- and 8.9-fold higher after stimulation with ATP and EGF/FGFb/B27, respectively (Fig. 1c, d). Likewise, α2δ1^+^ Huh7 cells also displayed higher Ca^2+^ oscillation frequency (Fig. 1e, f). Taken together, we conclude that Ca^2+^ oscillation is a signature that distinguishes liver CSCs from HCCs and nontumorigenic liver cells.

### Development of high-signal ER-targeted Ca^2+^ sensor GCaMP-ER2

**Fig. 2:**
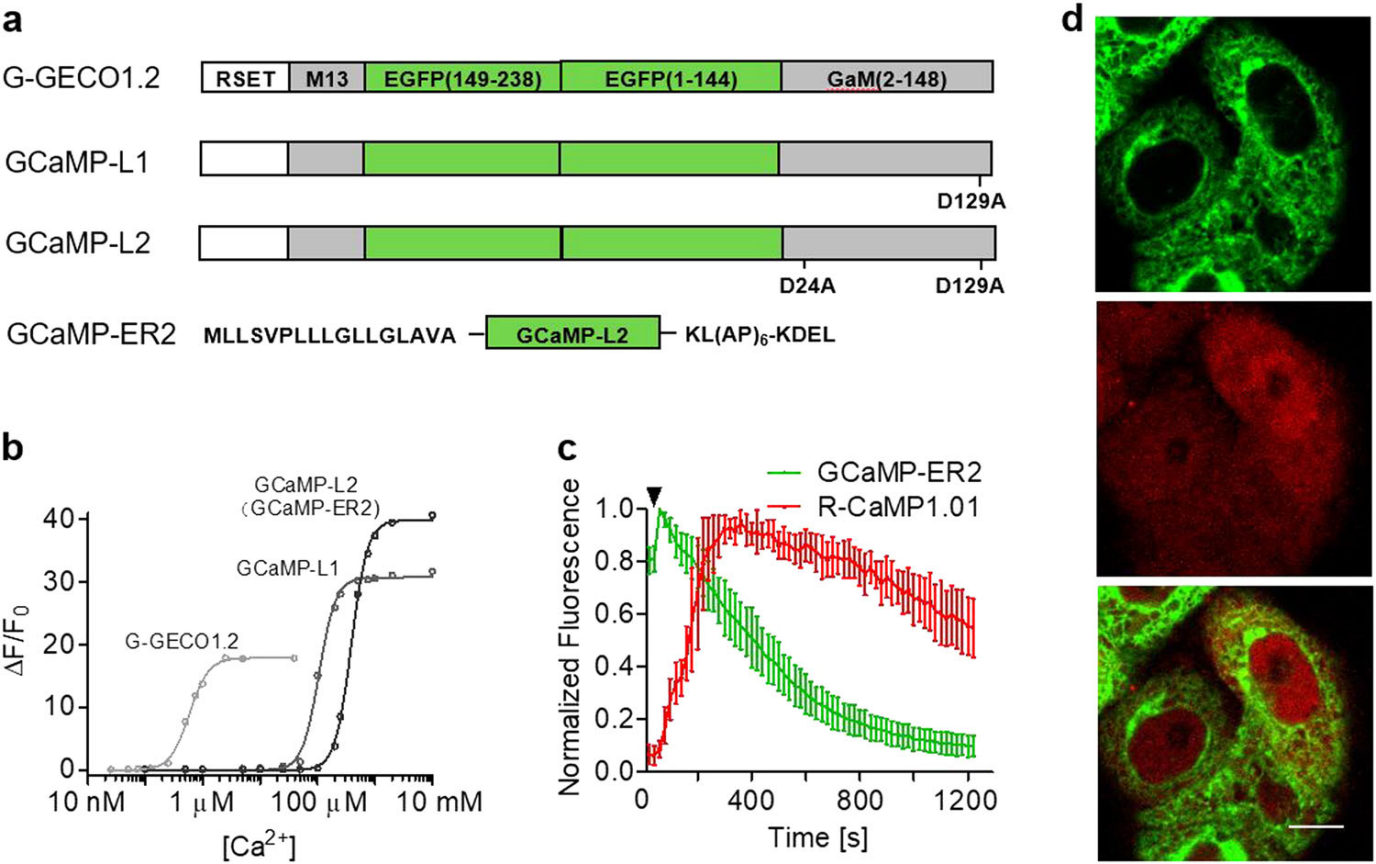
Engineering and characterization of ER localized Ca^2+^ sensor GCaMP-ER2. **a** Schematic structure of low affinity GCaMPs (GCaMP-L1 and GCaMP-L2) and ER-targeting of GCaMP-L2 (GCaMP-ER2). **b** In vitro Ca^2+^ titration of G-GECO1.2 and low affinity mutants GCaMP-L1 and GCaMP-L2. The *K*_d_ of GCaMP-L2 is 388 µM and the dynamic range (∆*F*_max_/*F*_0_) is 40. **c** Thapsigargin (Tg) induced depletion of Ca^2+^ in the endoplasmic reticulum. The ER Ca^2+^ and cytosolic Ca^2+^ response were observed by co-expression of GCaMP-ER2 and R-CaMP1.01 in HeLa cells. The arrowhead indicated time of 2 µM Tg stimulation. *n* = 9 cells. **d** Subcellular distribution of GCaMP-ER2 and R-CaMP1.01 in HeLa cells. R-CaMP1.01 is localized to the cytoplasm and nucleus (scale bar, 10 μm)

To measure ER Ca^2+^ dynamics, we developed GCaMP-ER2, a genetically encoded ER-targeting Ca^2+^ indicator with low Ca^2+^ affinity and high-dynamic range (*K*_d_ = 388 μM, Δ*F*_max_/*F*_0_ = 40). Briefly, we screened a series of random and structure–based mutations in the four calmodulin EF-hand loops of G-GECO1.2^13^, and identified a D129A mutant within the EF-hand of loop IV (GCaMP-L1, Fig. 2a), which has a dynamic range of 32 (∆*F*_max_/*F*_0_), and a *K*_d_ for Ca^2+^ of 106 μM. This variant was systematically mutated in EF-hand loops, resulting in an array of molecules with Ca^2+^ affinities ranging from 106 μM to 5.9 mM (Table S1). Figure 2a, b show the most promising ER sensor GCaMP-L2, with a dynamic range (∆*F*_max_/*F*_0_) of 40, a marked improvement over the previously highest signal strength ER reporter, GCaMPer/GCaMP3 (10.19)^19^ (∆*F*_max_/*F*_0_ = 14). As the *K*_d_ of GCaMP-L2 (388 μM) is well below the resting level of ER Ca^2+^, the indicator would be expected to operate near maximum brightness prior to ER Ca^2+^ release events.

Targeting of GCaMP-L2 to the ER was achieved by fusion of the N-terminal calreticulin ER targeting sequence MLLSVPLLLGLLGLAVA and the C-terminal ER retention signal KDEL. Addition of a KL(AP)_6_ linker between CaM and KDEL improved GCaMP-L2 brightness in HeLa cells, resulting in GCaMP-ER2 (Fig. 2a), which exhibited high fluorescence changes when thapsigargin was used to deplete ER Ca^2+^ (Fig. 2c). Figure 2d displays intracellularly compartmentalized signals in a HeLa cell recorded simultaneously with our newly developed GCaMP-ER2 and cytosol and nuclear localized RCaMP1.01.

### Distinctive ER Ca^2+^ dynamics in liver CSCs

**Fig. 3:**
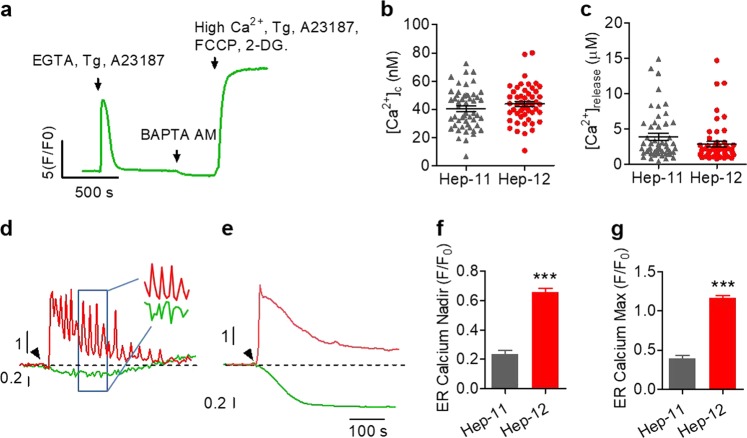
Distinct ER Ca^2+^ dynamics in cells displaying Ca^2+^ oscillation. **a**–**c** Measurements of the resting cytosolic Ca^2+^ level ([Ca^2+^]_c_) and the releasable ER Ca^2+^ store ([Ca^2+^]_release_) in Hep-12 and Hep-11 cells. **a** Representative trace and experimental protocol (details in Methods). **b** Scatter-plots of [Ca^2+^]_c_. **c** Scatter-plots of [Ca^2+^]_release_, an index of the Ca^2+^ content in the ER. *n* *=* 51 cells. **d**–**g** ER Ca^2+^ recovers and overshoots in Hep-12 cells displaying Ca^2+^ oscillation. **d**, **e** Representative time courses of cytosolic and ER Ca^2+^ dynamics in Hep-12 (**d**) and Hep-11 cells (**e**) upon 40 μM ATP stimulation. Dashed lines indicate the basal ER Ca^2+^ level. Inset shows an enlarged view of ER and cytosolic Ca^2+^ oscillations. **f**, **g** Statistics of the nadir **f** and maximal levels of ER Ca^2+^ during the 10 min of observation (**g**). Note the overshoot after initial depletion of ER Ca^2+^ in Hep-12 cells. *n* = 80 cells for Hep-12 and 54 for Hep-11. Data are shown as mean ± SEM (***P *<* 0.001 versus Hep-11 cells). All data were acquired with three independent experiments

Ca^2+^ oscillation is an emergent property of the entire cellular Ca^2+^-handling system. To investigate the cellular mechanism underlying the distinctive Ca^2+^ response of CSCs, we measured the resting cytosolic Ca^2+^ concentration ([Ca^2+^]_c_) and the capacity of the releasable ER Ca^2+^ store. The latter was indexed as the peak cytosolic Ca^2+^ concentration during release stimulated by the Ca^2+^ ionophore A23187 (10 μM) ([Ca^2+^]_release_) (Fig. 3a). We found that both [Ca^2+^]_c_ and [Ca^2+^]_release_ were similar in the two types of cells (Fig. 3b, c), excluding the possibility that Ca^2+^ oscillation in Hep-12 cells is brought about by a cytosolic or ER Ca^2+^ overload. Then we sought to measure ER Ca^2+^ dynamics. With the newly developed GCaMP-ER2 and Rhod-4, a cytosol-retained Ca^2+^ indicator, we simultaneously assessed the dynamic responses in both ER and cytosol (Fig. S2b). In Hep-11 cells, the ER Ca^2+^ store declined precipitously upon ATP stimulation, mirroring the rapid rise of the cytosolic Ca^2+^ transient (Fig. 3e). Subsequently, the GCaMP-ER2 fluorescence remained stable at a low level (nadir Δ*F*/*F*_0_ = −0.77) (Fig. 3f) even though the cytosolic Ca^2+^ level was returning toward an elevated steady-state level (Fig. 3f). This result suggests that the ER Ca^2+^ recycling mechanism is ineffective, presumably because of continued opening of the ER Ca^2+^ release channels. In Hep-12 cells, however, the initial ER Ca^2+^ depletion was smaller (nadir Δ*F*/*F*_0_ = −0.34) (Fig. 3d, f), and strikingly, it was rapidly restored and often followed by a conspicuous overshoot (peak *F*/*F*_0_ = 1.16) (Fig. 3d, g). The peak [Ca^2+^]_c_, however, was comparable in both types of cells (*F*/*F*_0_ = 3.68 ± 0.18 for Hep-11 and 3.23 ± 0.16 for Hep-12) (Fig. S2c, d). Close observation further revealed that each spiky cytosolic Ca^2+^ oscillation was accompanied by an antiphasic ER Ca^2+^ oscillation (Fig. 3d inset). This finding indicates that the cytosolic Ca^2+^ oscillation originates from periodic ER release and recycling. ER store recovery and overshoot were also found in the subpopulation of MHCC97-L cells with stimulated Ca^2+^ oscillation, whereas persistent ER Ca^2+^ depletion was associated with the population of cells lacking the oscillatory behavior (Fig. S2e). Taken together, we concluded that distinctive regulation of ER Ca^2+^ dynamics underlies the Ca^2+^ oscillation phenotype characteristic of liver CSCs.

### Role of ER Ca^2+^ cycling in the genesis of Ca^2+^ oscillation

**Fig. 4:**
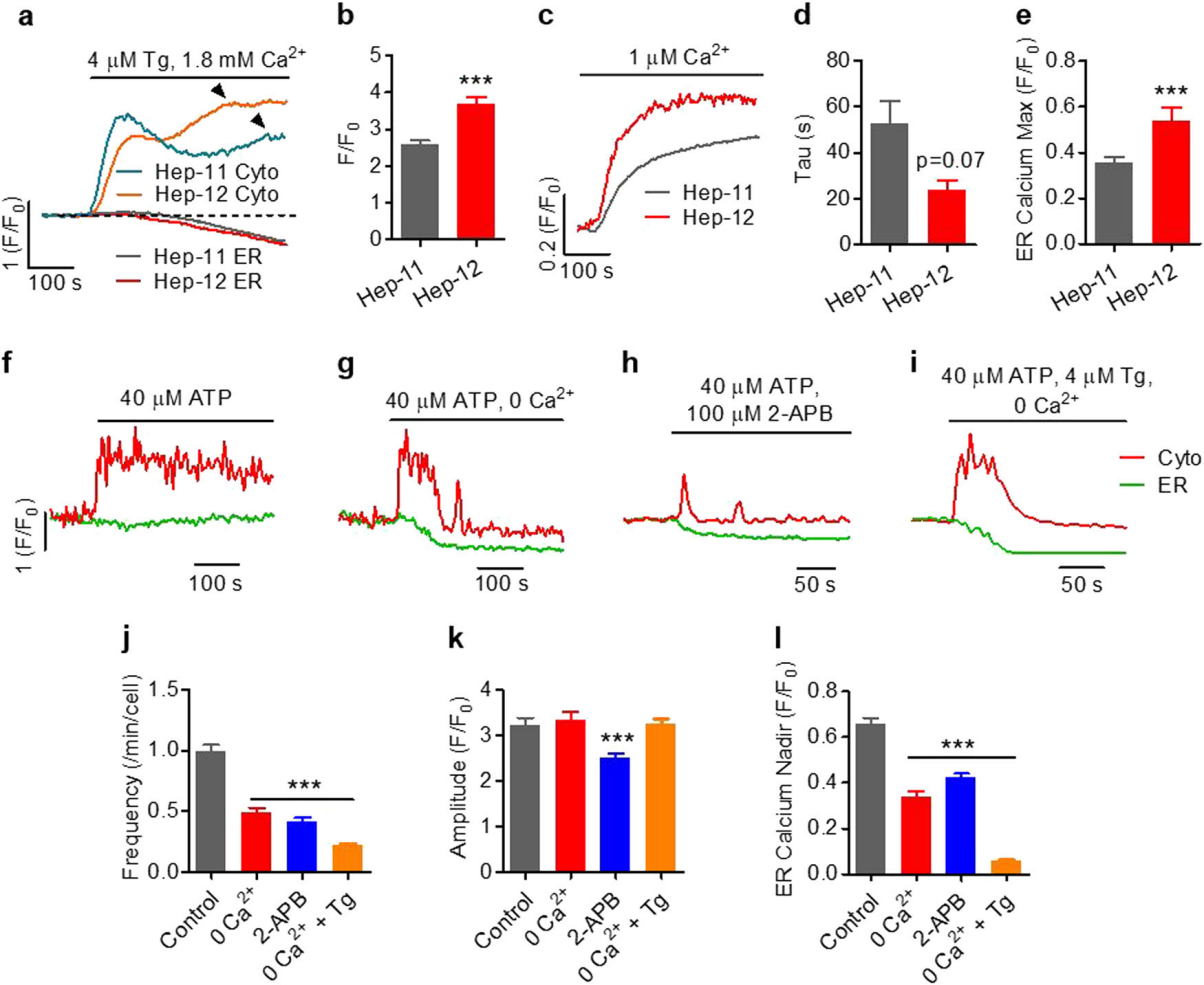
Identification of cellular determinants underlying Ca^2+^ oscillation. **a**, **b** SOCE in Hep-11 and Hep-12 cells. **a** Time courses of averaged cytosolic and ER Ca^2+^ transients in cells in which ER Ca^2+^ reuptake was inhibited with 4 μM Tg. Arrows mark the second peaks of cytosolic Ca^2+^ transients, reflecting the magnitude of SOCE. **b** Amplitudes of the second peaks (*n* = 62 cells for Hep-12 and 53 for Hep-11). **c**–**e** ER Ca^2+^ uptake in Hep-11 and Hep-12 cells. **c** Time courses of averaged ER Ca^2+^ changes after addition of 1 μM Ca^2+^. Cells were first permeabilized by 50 μg/mL saponin and ER Ca^2+^ was depleted by 4 μM Tg. **d** ER Ca^2+^ uptake time. **e** Maximal ER Ca^2+^ level during the 5-min observation (*n* = 38 cells for Hep-12 and 110 for Hep-11). **f**–**l** Role of SOCE and SERCA in Ca^2+^ oscillation in Hep-12 cells. Representative time courses of cytosolic and ER Ca^2+^ responding to 40 μM ATP without other treatment **f** or in conditions of (1) Ca^2+^-free solution without 4 μM Tg **g**; (2) addition of 100 μM 2-APB **h**; and (3) Ca^2+^-free solution with 4 μM Tg **i**. **j**–**l** Statistics of oscillation frequency **j**, first peak amplitude **k**, and the ER Ca^2+^ nadir **l** (*n* = 79 cells for control, 84 for 0 Ca^2+^, 95 for 100 μM 2-APB, and 117 for 0 Ca^2+^ and 4 μM Tg). Data are shown as mean ± SEM (***P *<* 0.001 versus Hep-11 **b**, **e** or control **j**–**l**). All data were acquired with three independent experiments

Store-operated Ca^2+^ entry (SOCE), the main Ca^2+^ entry pathway in nonexcitable cells, is activated upon depletion of the ER Ca^2+^ and plays pivotal roles in the timely replenishment of the internal Ca^2+^ store. In both cell types, inhibition of ER Ca^2+^ uptake by thapsigargin (4 μM) caused a biphasic cytosolic Ca^2+^ increase, with the second peak reflecting the SOCE capacity (Fig. 4a). It was clear that the Hep-12 cells displayed a greater SOCE component (Fig. 4b). In addition, faster ER Ca^2+^ uptake occurred in Hep-12 cells (Fig. 4c–e). To further delineate the specific role of SOCE, we measured cytosolic and ER Ca^2+^ dynamics in the absence of extracellular Ca^2+^. We found that the ATP-stimulated initial Ca^2+^ transients remained essentially unchanged; Ca^2+^ oscillation occurred in the early phase, but was quickly damped and eventually disappeared in 200 s (Fig. 4g). Concomitantly, the ER Ca^2+^ store showed a general trend of depletion without replenishment (Fig. 4g, l). Application of 100 μM 2-aminoethoxydiphenyl borate (2-APB), an inhibitor of both SOCE and IP_3_Rs at this concentration, greatly attenuated the Ca^2+^ oscillation and prevented the ER store from recovering (Fig. 4h, j, l). The initial Ca^2+^ transient was also markedly smaller and briefer (Fig. 4k), perhaps because of partial inhibition of release. Simultaneous inhibition of Ca^2+^ entry and ER Ca^2+^ recycling (0 Ca^2+^ and 4 μM thapsigargin) had additive effects (Fig. 4i, j, l). These results indicate that the persistence of Ca^2+^ oscillation is attributable to the rapid refilling and overshoot of the ER store due to enhanced SOCE and ER Ca^2+^ uptake. Nonetheless, they are not obligatory for the genesis of stimulated Ca^2+^ oscillation; rather, they play a permissive role in preventing it from damping.

### IP_3_R2 as the intrinsic oscillator underlying Ca^2+^ dynamics in Hep-12 cells

**Fig. 5:**
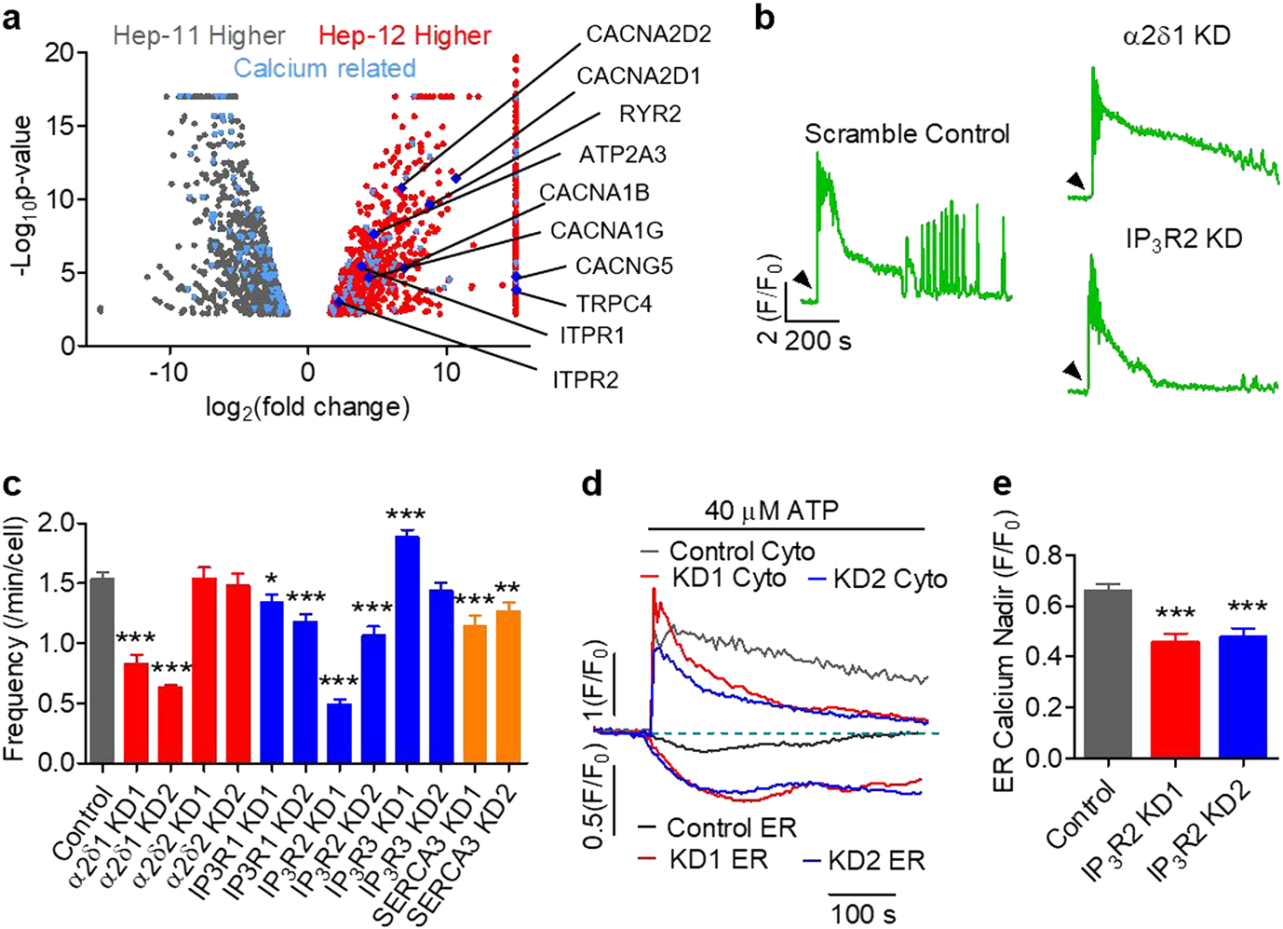
Identification of key Ca^2+^ regulators pivotal to Ca^2+^ oscillation. **a** Volcano plots of mRNA level differences in Hep-12 and Hep-11 cells. Light blue dots mark calcium regulators and Deep blue dots represent specific direct Ca^2+^-handling molecules. **b**, **c** Effects of knockdown of Ca^2+^ regulators identified in (**a**) on ATP (40 μM)-stimulated Ca^2+^ oscillation. **b** Representative Ca^2+^ dynamics after α2δ1 and IP_3_R2 knockdown. Arrows mark time of ATP stimulation. **c** Statistics of oscillation frequency (*n* > 100 cells; data are shown as mean ± SEM; *P < 0.05 versus scrambled control, **P < 0.01, ***P *<* 0.001). **d**, **e** During 40 μM ATP treatment, ER Ca^2+^ continued to drop in Hep-12 cells with IP_3_R2 knockdown. **d** Time courses of averaged cytosolic Ca^2+^ and ER Ca^2+^ of IP_3_R2-knockdown Hep-12 cells responding to 40 μM ATP. **e** Statistics of ER Ca^2+^ nadir (*n* = 80 cells in control, 37 for IP_3_R2 KD1, and 37 for IP_3_R2 KD2). All data were acquired with three independent experiments

Next, we sought to identify the key molecular players underlying the Ca^2+^ oscillation phenotype. By RNA-sequencing analysis, we identified 1887 genes expressed differently in Hep-12 versus Hep-11 cells (Fig. 5a). Among them, 175 genes were found in Ca^2+^-related Gene Ontology (1093 genes included) (Fig. 5a). Interestingly, of the ten genes that directly participate in Ca^2+^ transport, all were significantly upregulated in Hep-12 cells (Fig. 5a). These genes code proteins for pore subunits of the N-type (α1B) and T-type (α1G) Ca^2+^ channels, subunits associated with most voltage-operated Ca^2+^ channels (VOCCs) (α2δ1, α2δ2, and γ5), ER Ca^2+^ release channels (IP_3_R1, IP_3_R2, and RyR2), and the ER Ca^2+^ uptake pump (SERCA3), as well as transient receptor potential TRPC4 channels. Notably, α2δ1, which helps to anchor VOCCs in the plasma membrane, has been previously reported by us as a functional liver CSC marker^6,7^.

To identify key Ca^2+^ regulators, we targeted the top five genes on the list, CACNA2D1, CACNA2D2, ITPR1, ITPR2, and ATP2A3, and determine the consequences of the Ca^2+^ oscillation phenotype. We used α2δ1 as a positive control on some occasions, and included ITPR3 (IP_3_R3 gene) alongside ITPR1 (IP_3_R1 gene) and ITPR2 (IP_3_R2 gene) to acquire a comprehensive view of the IP_3_R family. We established stable cell lines harboring shRNAs against these genes (Fig. S3a). Functional analysis revealed that Ca^2+^ oscillation frequency was severely suppressed after knockdown of α2δ1 and IP_3_R2, modestly decreased with knockdown of IP_3_R1 and SERCA3, and largely unchanged after knockdown of α2δ2 and IP_3_R3 (Fig. 5b, c, Fig. S3b). The amplitude of the first peak, which reflects the ER Ca^2+^ content as well as fractional release, was mildly decreased (knockdown of IP_3_R1, IP_3_R2, or IP_3_R3) or unchanged (knockdown of α2δ1, α2δ2, or SERCA3) (Fig. S3c). These results show that multiple Ca^2+^ regulators orchestrate the phenotype of Ca^2+^ oscillation. Nonetheless, other than α2δ1, IP_3_R2 emerged as the most important molecular determinants. Particularly, after IP_3_R2 knockdown, the ER Ca^2+^ phenotype was drastically altered, with greater initial release and without any recovery (Fig. 5d, e), resembling those in non-oscillatory HCCs. That is, IP_3_R2 may serve as an intrinsic oscillator essential for Ca^2+^ oscillation. Previous studies have also shown that, unlike IP_3_R1 and IP_3_R3, IP_3_R2 is prone to generate the Ca^2+^ oscillation behavior^28–30^.

### Ca^2+^ oscillation frequency positively correlates with spheroid-forming efficiency in Hep-12 cells

**Fig. 6:**
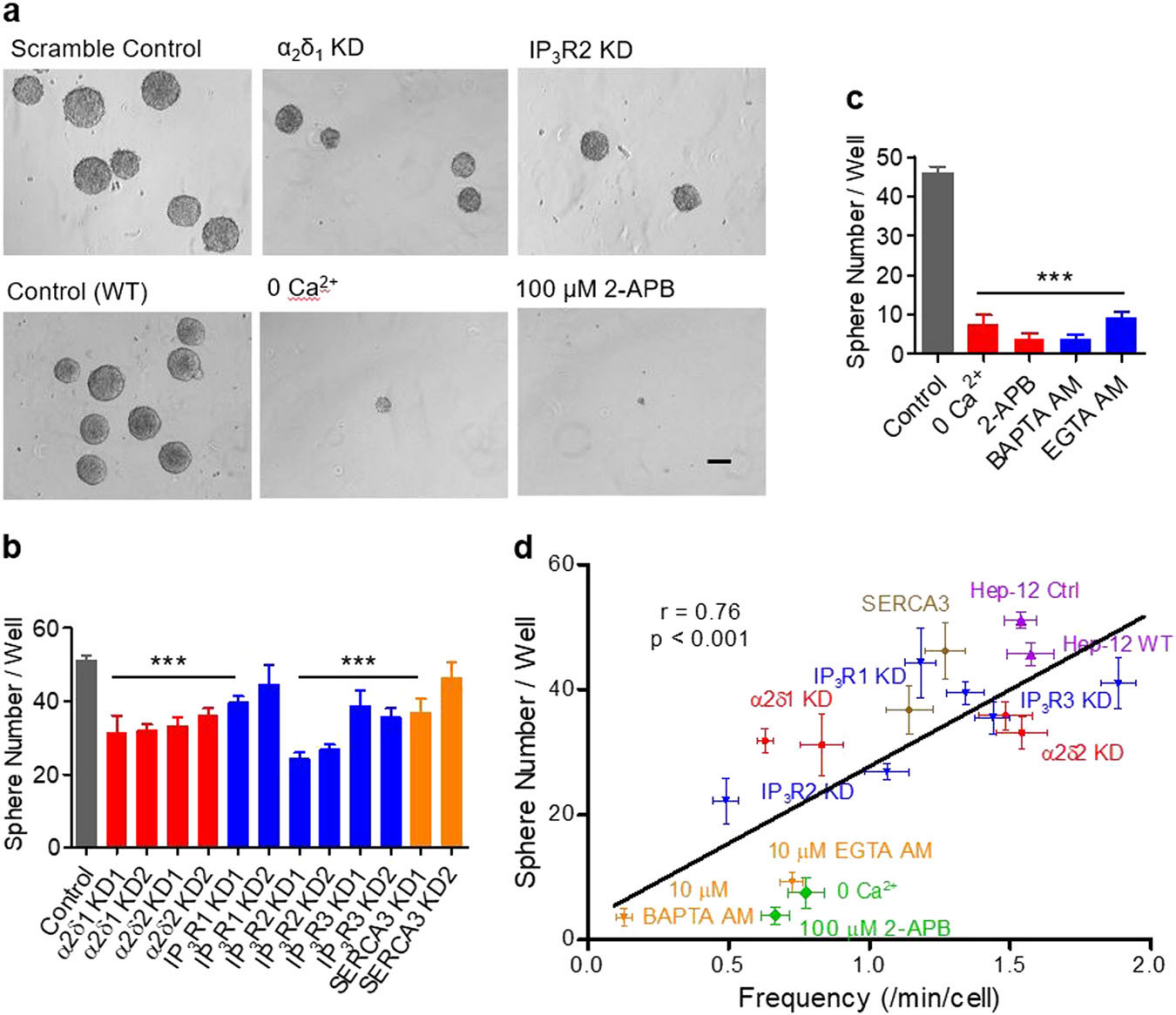
Ca^2+^ oscillation frequency positively correlates with spheroid-forming efficiency. **a** Phase-contrast images of spheroids formed by Hep-12 cells under different conditions (scale bar: 100 μm). **b, c** Statistics of spheroid-forming efficiency. **b** Scrambled control group and shRNA knockdown groups. **c** Groups with and without drug treatment. One hundred cells per well were plated. Spheroids (*R* > 100 μm) were counted under a stereomicroscope (*n* = 6–12; ***P < 0.001 versus control). **d** Scatter-plot of oscillation frequency versus spheroid-forming efficiency. Linear regression (solid line) yielded a positive correlation coefficient (*r*) of 0.76 (*P* < 0.001). Data are expressed as mean ± SEM, *n* *=* 18. All data were acquired with at least three independent experiments

To determine the functional significance of Ca^2+^ oscillation in CSC biology, we assessed the efficiency of spheroid formation, which reflects the self-renewal capacity, in relation to Ca^2+^ oscillation frequency over a broad range of experimental conditions. The experimental groups included: (i) knockdown of the VOCC subunits α2δ1 and α2δ2; (ii) knockdown of the ER release channels IP_3_R1, IP_3_R2, and IP_3_R3; (iii) removal of extracellular Ca^2+^; (iv) inhibition of SOCE; and (v) treatment with BAPTA-AM or EGTA-AM to retard Ca^2+^ changes. We found that, while inhibiting Ca^2+^ oscillation, both α2δ1 and α2δ2 knockdown significantly compromised the self-renewal (Fig. 6a, b, Fig. S4a), confirming and extending our previous report^6^. Spheroid formation was also reduced when extracellular Ca^2+^ was removed, SOCE was inhibited or cytosolic Ca^2+^ was buffered (Fig. 6a, c, Fig. S4a). Notably, of the three IP_3_R subtypes, knockdown of IP_3_R2, but not IP_3_R1 and IP_3_R3, greatly curtailed the spheroid formation (Fig. 6a, b, Fig. S4a).

From these data, a general pattern emerged in which lowering the Hep-12 Ca^2+^ oscillation frequency effectively decreased the efficiency of spheroid formation, regardless of whether the type of Ca^2+^ manipulation was genetic, biophysical, or pharmacological. For quantitative analysis, we summarized our data in a scatter-plot (Fig. 6d). Linear regression revealed a strong positive correlation between spheroid-forming efficiency and Ca^2+^ oscillation frequency (*r* = 0.76, *p* < 0.001) (Fig. 6d). This result strongly suggests that the signature Ca^2+^ oscillation in liver CSCs plays an important functional role in promoting their self-renewal.

### Targeting IP_3_R2-mediated Ca^2+^ oscillation suppresses tumor formation of liver CSCs

**Fig. 7:**
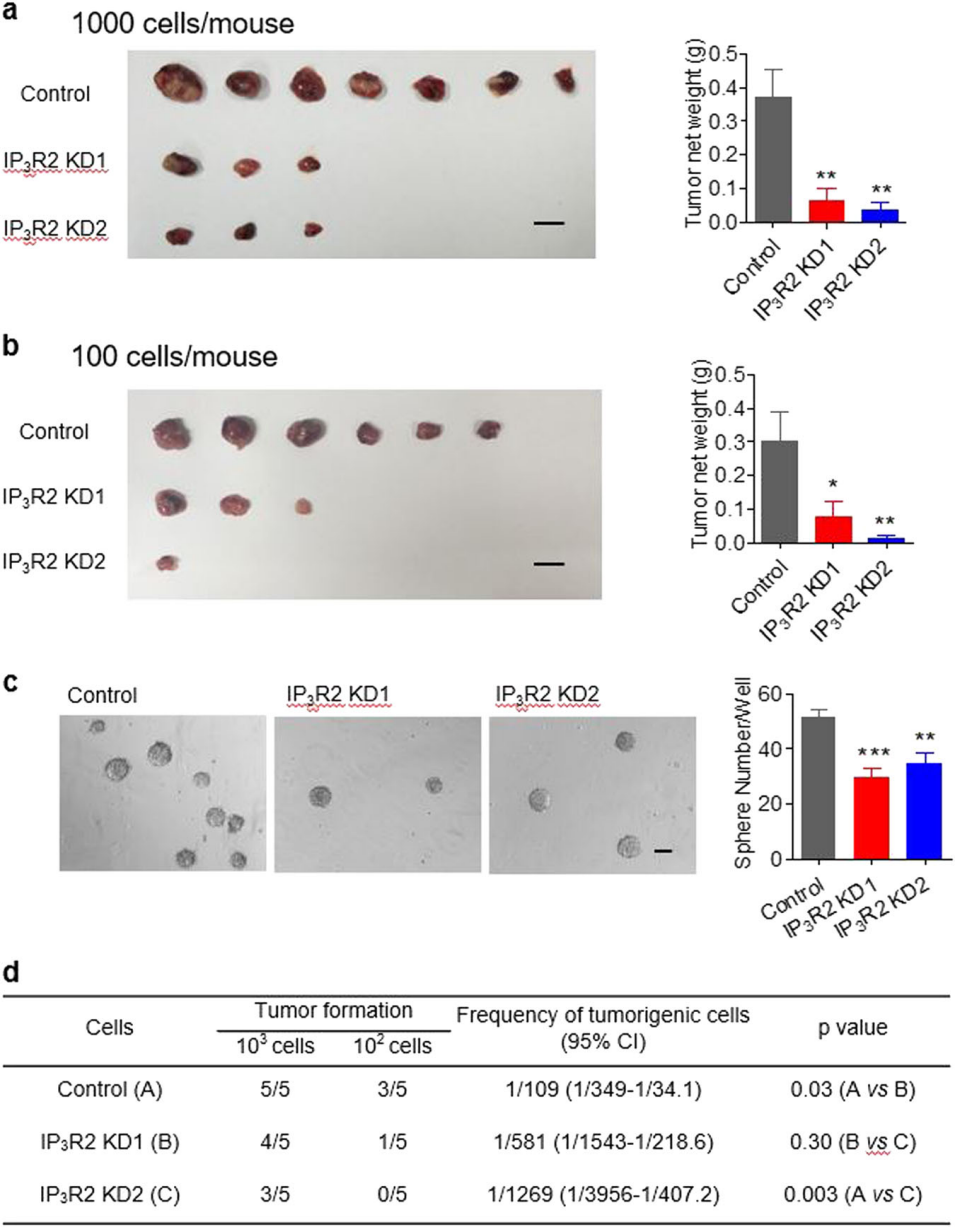
Crucial role of IP_3_R2 in self-renewal of liver cancer stem cells. **a**, **b** In vivo tumorigenicity of IP_3_R2-knockdown Hep-12 cells. One thousand (**a**) or one hundred cells (**b**) per mouse were injected (*n* = 7). Left: Images showing dissected tumors at termination of the experiment time (scale bars: 1 cm). Right: Tumor weight. Data are shown as mean ± SEM (*P < 0.05 versus control, ***P < 0.001). **c** IP_3_R2 knockdown suppresses self-renewal of α2δ1^+^ Huh7 cells. Left: Phase-contrast images of spheroid formation. Right: Statistics of spheroid-forming efficiency. One hundred cells per well were plated. Spheroids (*R* > 100 μm) were counted under a stereomicroscope (*n* = 9; **P < 0.01 versus control, ***P < 0.001. **d** Tumorigenic cell frequency in α2δ1^+^ Huh7 cells with IP_3_R2 knockdown in NOD/SCID mice

In light of the newly established relationship between Ca^2+^ oscillation and spheroid formation, we hypothesized that targeting the Ca^2+^ oscillation machinery might provide an effective therapeutic strategy to limit CSC expansion and therefore retard or even prevent the genesis of tumors. Among the aforementioned molecular participants in the genesis and regulation of Ca^2+^ oscillation, IP_3_R2 is particularly noteworthy, not only because it is greatly upregulated in liver CSCs, but also its cyclic opening may serve as the intrinsic oscillator^28–30^. To further evaluate the impact of IP_3_R2 on the self-renewal of Hep-12 cells, we injected 10^3^ or 10^2^ scrambled control cells and IP_3_R2-knockdown cells subcutaneously into nonobese diabetic/severe combined immunodeficient (NOD/SCID) mice to test their tumorigenicity potential. Tumors were dissected and weighed 42 or 52 days after injection of 10^3^ or 10^2^ cells, respectively. As shown in Fig. 7a, b, the tumorigenicity of Hep-12 cells was significantly suppressed by IP_3_R2 knockdown. To further confirm the role of IP_3_R2, we purified α2δ1^+^ CSCs from Huh7 cells and assayed spheroid formation and tumorigenicity with IP_3_R2 knockdown. Both the efficiency of spheroid formation and the frequency of tumorigenic cells were markedly inhibited (Fig. 7c, d), substantiating the pivotal role of IP_3_R2 in the self-renewal of liver CSCs.

## Discussion

In this study, we demonstrate that Ca^2+^ oscillation is a functional signature of liver CSCs, and provide evidence that targeting Ca^2+^ oscillation can effectively limit the self-renewal of liver CSCs and thereby tumor initiation and progression. In response to a panel of niche factors (ATP, EGF/FGFb/B27, and IL-6), robust Ca^2+^ oscillation occurred in liver CSCs, including CSC-enriched Hep-12 cells, the small portion of α2δ1^+^ Hep-11 cells, and α2δ1^+^ Huh7 cells. This Ca^2+^ phenotype is CSC-specific because the vast majority of matching Hep-11 and Huh7 cancer cells did not show similar Ca^2+^ oscillation, and neither did HCC LM3, MHCC97-L, and the immortalized liver cell line MIHA. This finding is in general agreement with previous reports that Ca^2+^ oscillation occurs in a portion of highly metastatic, but not in weakly/non-metastatic human prostate and breast cancer cells^31^. Such CSC Ca^2+^ oscillation plays important roles in cancer biology. The efficiency of spheroid formation was significantly decreased by suppressing Ca^2+^ oscillation by removing extracellular Ca^2+^, inhibiting SOCE, buffering cytosolic Ca^2+^, or knocking down key Ca^2+^ regulators (IP_3_R2 or α2δ1). Strikingly, there was a positive correlation between the Ca^2+^ oscillation frequency and spheroid formation efficiency under a broad range of conditions. More importantly, we showed that CSCs with defective Ca^2+^ oscillation, i.e., knockdown of IP_3_R2, exhibited a greatly compromised ability to form tumors in NOD/SCID mice.

Ca^2+^ oscillation reflects a system-level, emergent behavior of the complex Ca^2+^-handling machinery. To sustain a persistent cyclic Ca^2+^ oscillation, the ER store must be kept replenished. With our newly developed ER Ca^2+^ sensor GCaMP-ER2, we disclosed a surprising new insight at the cellular level. In CSCs undergoing niche factor-stimulated Ca^2+^ oscillation, initial ER depletion was followed by a complete restoration and oftentimes an overshoot of the ER Ca^2+^ content. In HCCs displaying no Ca^2+^ oscillation, however, stimulated ER Ca^2+^ release was followed by a deep ER depletion without any sign of recovery over the period of observation (15 min). The distinctive ER Ca^2+^ dynamics are in part attributable to higher activity of SOCE and ER Ca^2+^ recycling in CSCs (this study) as well as Ca^2+^ entry through VOCCs as we reported previously^6^. More importantly, these results strongly suggest that the ER release channels in CSCs undergo cyclic, brief openings, allowing for ER Ca^2+^ retention and supporting the Ca^2+^ oscillation behavior, whereas those in HCCs may be kept open after stimulation, preventing replenishment of the ER Ca^2+^. Through RNA-seq and bioinformatics analysis confirmed by functional validation, we identified the ER release channel IP_3_R2 as the intrinsic oscillator driving the CSC Ca^2+^ phenotype, while the other Ca^2+^ proteins examined serve largely permissive roles (e.g., α2δ1 and SERCA3).

The IP_3_R family consists of three subtypes, IP_3_R1, IP_3_R2, and IP_3_R3, which share 60–80% homology of amino acid sequence, and the subtype expression is differentially regulated in response to physiological and pathological stimuli. There is a bell-shaped Ca^2+^-dependence of the channel activity: activation via the CICR mechanism at low [Ca^2+^]_*i*_, inhibition via Ca^2+^-dependent inactivation at higher [Ca^2+^]_*i*_^32^. These properties lay the foundation for Ca^2+^ oscillation in nonexcitable cells. All three IP_3_R subtypes are similar in terms of permeability, but their high-Ca^2+^ inactivation properties are variable^33^. Functional experiments have shown that IP_3_R2 is required for long-lasting and regular Ca^2+^ oscillation^28–30^, IP_3_R1 is responsible for short and irregular Ca^2+^ oscillation^28^, and IP_3_R3 produces a monophasic Ca^2+^ spike^28^ and even has anti-oscillatory actions^28,34^. More interestingly, IP_3_R2 co-expression with IP_3_R1 or IP_3_R3 facilitates Ca^2+^ oscillation^28^. In this regard, we found that IP_3_R2 was specifically expressed in liver CSCs but not HCCs; and knockdown of IP_3_R2, but not IP_3_R1 and IP_3_R3, repressed Ca^2+^ oscillation (Fig. 5b, c, Fig. S3b), and hence the capacity for self-renewal in CSCs (Fig. 6a, b, Fig. S4a). Knockdown of IP_3_R2 also caused ER Ca^2+^ phenotypes similar to that in a typical Hep-11 cell. Collectively, these data indicate that IP_3_R2 is a target of choice for limiting CSC self-renewal through manipulating Ca^2+^ oscillation. Indeed, CSCs with knockdown of IP_3_R2 showed depressed sphere formation (Figs. 6a, b, 7c) and in vivo tumorigenesis (Fig. 7a, b, d).

CSCs rely on niches to maintain their stemness and prompt metastasis^35^. In this regard, we found that all niche factors examined were able to elicit robust Ca^2+^ oscillation in liver CSCs, supporting the hypothesis that Ca^2+^ signaling is at the root of CSC maintenance and survival. Ca^2+^ oscillation can mediate cellular signaling in a frequency-modulatory mode, with the frequency varying from tens of Hz to tens of mHz. Many protein kinases, protein phosphatases, and transcription factors are known to decipher Ca^2+^ oscillation, and each has a specific dependence on oscillation frequency, interestingly with little overlap^36^. Future investigations to determine the involvement of these decoder proteins in CSC Ca^2+^ oscillation to signal self-renewal are warranted.

In summary, we have shown that niche factor-stimulated Ca^2+^ oscillation is a signature feature pivotal to self-renewal of liver CSCs. Further, through the development of a new genetically coded ER Ca^2+^ sensor GCaMP-ER2, we have identified IP_3_R2, which is enriched in CSCs, as the intrinsic oscillator driving such Ca^2+^ oscillation. Targeting Ca^2+^ oscillation in general and IP_3_R2 in particular might afford a physiologically inspired anti-tumor strategy by uprooting CSCs to limit tumor initiation, progression, recurrence, and drug resistance.

## Materials and methods

### Cell culture and establishment of stable target gene-knockdown cell lines

Hep-12, Hep-11, and Huh7 cells^37^ were cultured in RPMI 1640 medium (Invitrogen) supplemented with 10% fetal bovine serum (FBS) (Invitrogen) at 37 ℃ under 5% CO_2_. The LM3 and MHCC97-L cell lines were gifted by Dongqin Yang (Huashan Hospital, Fudan University), and the MIHA cell line was gifted by Jinying Ning (Crownbio Co., Beijing, China). The HeLa cell line was kept in our labs. All four cell lines were cultured in Dulbecco’s modified Eagle’s medium (Invitrogen) supplemented with 10% FBS. To generate stable Hep-12 cell lines with knockdown of target genes, packaging plasmids pLP1, pLP2, and pVSVG (Invitrogen) were transfected with expression plasmids harboring shRNA targeting the specific genes into 293FT cells at the ratio 1:1:1:3, then the lentivirus was collected and cells were infected. The puromycin-resistant cells were cultured and stable target gene-knockdown cell lines were acquired. ShRNAs (Sigma) were gifted by Guoqiang Bi (University of Science and Technology of China) and their sequences are listed in Table S2.

### Calcium measurement and imaging

Fluo-4 AM (Invitrogen) was used to monitor cytosolic Ca^2+^ dynamics. When ER Ca^2+^ was measured simultaneously using GCaMP-ER2, Rhod-4 AM (AAT Bioquest) was used for cytosolic Ca^2+^ detection. Briefly, cells were incubated in the presence of Fluo-4 AM (5 μM) or Rhod-4 AM (5 μM) at room temperature for 30 min. After washing with Tyrode’s solution consisting of (in mM) 137 NaCl, 5.4 KCl, 1.2 MgCl_2_, 1.2 NaH_2_PO_4_, 1.8 CaCl_2_, 10 glucose, and 20 HEPES (pH 7.35, adjusted with NaOH), Fluo-4 or Rhod-4 fluorescence was measured with a Zeiss LSM 710 confocal microscope equipped with a 40×, 1.3 NA oil-immersion objective. GCaMP-ER2 and RCaMP1.01 plasmids were co-transfected into HeLa cells using Lipofectamine 2000 (Invitrogen) 48–72 h before imaging and measurement. For ER Ca^2+^ measurement in CSCs and HCCs, adenovirus expressing GCaMP-ER2 was used to infect the cells 48 h before measurement. For Fluo-4 fluorescence measurement, cells were excited at 488 nm and emission collected at 493–622 nm. For GCaMP-ER2 and Rhod-4 dual indicator measurement, images were captured in multi-track mode with excitation at 488 or 543 nm and emission collection at 490–516 or 556–733 nm, respectively.

The cytosolic Ca^2+^ level was quantified using the equation [Ca^2+^] = *K*_d_ (*F* − *F*_min_)/(*F*_max_ − *F*). After measurement of fluorescence at rest (*F*_0_), cells were exposed to Tyrode’s solution (zero Ca^2+^) with 4 mM EGTA, 5 μM thapsigargin, and 10 μM A23187. Store Ca^2+^ release was induced and cytosolic Ca^2+^ increased abruptly and reached the peak in ~10 s (*F*_release_) followed by a gradual decline. When stabilized, 100 μM BAPTA-AM was added to further chelate cytosolic Ca^2+^ to measure the minimum fluorescence level (*F*_min_). Then, for measurement of the maximal level (*F*_max_), cells were exposed to Tyrode’s solution containing 10 mM Ca^2+^, 5 μM thapsigargin, 12 μM A23187, 3 μM FCCP, and 20 mM 2-DG. Assuming that *K*_d_ = 1 μM for Fluo-4 in intact cells^38^, the resting Ca^2+^ [Ca^2+^]_c_, and the releasable store Ca^2+^ indexed by [Ca^2+^]_release_ were obtained with the above equation.

### Synthesis of low-affinity GCaMP (GCaMP-L) mutants

Mutations of CaM were introduced into the G-GECO1.2 sequence by overlap extension PCR. The amplified product was inserted into the *Nhe*I/*Hind*III sites of pRSET-A expression vector (Invitrogen). The mutant proteins were expressed in *E. coli* BL21 Star (DE3) pLysS cells and purified using Ni-charged resins as previously described^39^. After elution, the buffer was changed to 30 mM MOPS (pH 7.2) with 100 mM KCl using an Amicon Ultra-4 filter unit (Millipore). Protein concentration was measured using BCA Protein Assay (Pierce).

### In vitro characterization of purified proteins

Calcium titration of G-GECO1.2 was performed by Calcium Calibration Buffer Kit #1 (Invitrogen). For calcium titration of low affinity mutants, a series of zero to 10 mM [Ca^2+^]_free_ buffer were made in 1 mM EGTA, 50 mM MOPS, and 100 mM KCl (pH 7.2) and [Ca^2+^]_free_ concentrations were calculated using WEBMAXC EXTENDED program (maxchelator.stanford.edu). The fluorescence of 1 µM purified protein in various [Ca^2+^]_free_ buffers were measured with excitation at 485/20 nm and emission at 516/20 nm using a Synergy 2 Microplate Reader (Biotek).

### Construction of ER-targeted GCaMP-ER2

The GCaMP-L2 was targeted to and retained in the ER via the N-terminal calreticulin ER targeting sequence MLLSVPLLLGLLGLAVA and the C-terminal ER retention signal KDEL, respectively, with a linker KL(AP)_6_ between CaM and retention signal. The final construct was generated by PCR with primers containing described coding sequences and GCaMP-L2 template. The PCR product was cloned into the pEGFP-N1 mammalian expression vector (replacing EGFP) using *Bgl*II and *Not*I restriction sites.

### Cell labeling and flow cytometry

The α2δ1 antibody (Abcam) was directly labeled with the Lightning-Link PE-Cy5 Labeling Kit following the vendor’s protocol (Innova Biosciences). For flow cytometry, cells were digested, dispersed, labeled, and analyzed as previously described^40^.

### Sphere formation assay

Cells were plated in ultra low attachment 96-well plates (Corning) and cultured in Dulbecco’s modified Eagle’s medium/F12 (Invitrogen) supplemented with B27 (Invitrogen), 40 ng/ml epidermal growth factor (Invitrogen), 40 ng/ml basic fibroblast growth factor (Peprotech), and 1% methylcellulose (Sigma). Cells were incubated for 2–3 weeks, and spheres were counted under a stereomicroscope (Olympus).

### Tumorigenicity assay in NOD/SCID mice

Cells were suspended in 50 μL of a 1:1 mixture of plain RPMI 1640 and Matrigel (BD Biosciences) and injected subcutaneously into the back of 4- to 6-week-old NOD/SCID mice (Vital River). Tumor formation was monitored weekly.

To assess the tumorigenicity detection of α2δ1^+^ Huh7 cells with IP_3_R2 knockdown, sorted cells were first infected with shRNA-IP_3_R2 or scrambled control lentivirus for 4 h at 37 ℃.

All animals were treated in compliance with the Guide for the Care and Use of Laboratory animals published by the US National Institutes of Health (NIH Publication No. 85-23, revised 1996) and approved by the Animal Care and Use Committee of Peking University (accredited by AAALAC international).

### RNA sequencing (RNA-seq) analysis

Using TRIzol reagent (Invitrogen), total RNA was isolated from cells according to the manufacturer’s protocol. The quality of the extracted RNA was controlled using Agilent 2100. A sequencing library was prepared using the NEBNext^®^Ultra^TM^ RNA Library Prep Kit for Illumina^®^ (New England Biolabs) following the manufacturer’s recommendations. Deep sequencing was then performed on an Illumina Hiseq 4000 platform and 150-bp paired-end reads were generated. To obtain the differentially-expressed genes, RNA-seq reads were first aligned to the human genome (hg19) with TopHat (v 1.2.0) and all the mapping results were evaluated as previously described^41^. Cuffdiff software (v 2.0.2) was used to obtain differentially expressed genes with a cutoff of fold-change > 2 and *q* value < 0.05. By searching Gene Ontology (http://www.geneontology.org/) we found Ca^2+^-related genes distributed in process, function, and component.

### Western blotting

Cells lysates were obtained by incubating cells directly with sodium dodecyl sulphate polyacrylamide gel electrophoresis (SDS-PAGE) loading buffer. After ultrasonicating 5 times (5 s each), lysates were heated at 100 °C for 10 min. Proteins were separated on 6% SDS-PAGE gel (for IP_3_R expression) or 8% SDS-PAGE gel (for α2δ1, α2δ2, and SERCA3 expression) and transferred to a 0.45-μm polyvinylidene difluoride membrane (Millipore). Membranes were blocked with 5% bovine serum albumin (for IP_3_R expression) or 5% nonfat dry milk (for α2δ1, α2δ2, and SERCA3 expression) and incubated with primary antibody overnight at 4 °C. Primary antibodies against IP_3_R1 (Abcam, 1:500), IP_3_R2 (Millipore, 1:50), IP_3_R3 (BD Biosciences, 1:1000), α2δ1 (Abcam, 1:1000), α2δ2 (Sigma, 1:2000), SERCA3 (Abcam, 1:500), and tubulin (Sigma-Aldrich, 1:2000) were used.

### Statistics

The data are expressed as the mean ± SEM and, when appropriate, Student’s *t* test was applied to determine statistical significance. *P* < 0.05 was considered statistically significant.

## Supplementary information

Supplementary materials

Supplementary Figure 1

Supplementary Figure 2

Supplementary Figure 3

Supplementary Figure 4

Supplementary Figure 5

Supplementary Table 1

Supplementary Table 2

Supplementary Movie 1

Supplementary Movie 2
