# Supplementary material for: Central role of IP3R2-mediated Ca2+ oscillation in self-renewal of liver cancer stem cells elucidated by high-signal ER sensor
Source: Cell Death Dis. 2019 May 21;10(6):396. doi: 10.1038/s41419-019-1613-2 (PMC6529459; doi:10.1038/s41419-019-1613-2)
Supplement: Supplementary file 1 — Supplementary materials [file 41419_2019_1613_MOESM1_ESM.docx]

**Central Role of IP_3_R2-mediated Ca^2+^ Oscillation** **in Self-Renewal of Liver Cancer Stem Cells Elucidated by High Signal ER Sensor**

***Sun et al***

**Supplementary Materials**

**Supplementary Figure 1. Time course and frequency analysis of stimulated Ca^2+^ oscillation.** (**a**) Representative Ca^2+^ oscillation evoked by 40 μM ATP in a Hep-12 cell. Diamonds mark individual oscillations. (**b**) Statistics of percentage of cells showing Ca^2+^ oscillation in groups in Figure 1a.

**Supplementary Figure 2. Distinctive ER Ca^2+^ dynamics associated with the Ca^2+^ oscillation phenotype.** (**a**) Brightness and dynamic range of low affinity GCaMP (GCaMP-L) mutants. All mutants listed contain CaM D129A mutation in G-GECO1.2. Baseline brightness were normalized to fluorescence of G-GECO1.2 at zero [Ca^2+^]. F_max_ were measured at 10 mM saturating [Ca^2+^], except fluorescence of D20A, D56A and D93A which were measured at 100 mM saturating [Ca^2+^]. F_min_ is fluorescence intensity at zero [Ca^2+^]. GCaMP-L2 (D24A/D129A mutant) has a highest dynamic range, F_max_/F_min_ = 41. (**b**) Confocal images of cells expressing GCaMP-ER2 and stained with Rhod-4 AM (scale bar, 5 μm). (**c**) Averaged time courses of cytosolic and ER Ca^2+^ of Hep-12 cells and Hep-11 cells upon treatment with 40 μM ATP. (**d**) Statistics of the first peak amplitude of the cytosolic Ca^2+^ response (*n* = 80 cells for Hep-12 and 54 for Hep-11). Data are acquired with three independent experiments and shown as mean ± SEM. (**e**) Representative time courses of cytosolic and ER Ca^2+^ in MHCC97-L cells responding to 40 μM ATP. Arrowheads mark time of ATP stimulation. Note the ER Ca^2+^ quickly recovers and then overshoots after initial depletion in cells displaying the oscillatory Ca^2+^ phenotype, and the sustained ER depletion in cells displaying no prominent oscillatory behavior.

**Supplementary Figure 3. Ca^2+^ dynamics after knockdown of targeted genes.** (**a**) Western blots of specific protein expression in Hep-12 cells stably infected with lentivirus harboring scrambled shRNA or shRNA targeting α2δ1, α2δ2, IP_3_R1, IP_3_R2, IP_3_R3, or SERCA3. (**b**) Representative traces are shown with arrows marking the onset of ATP stimulation. (**c**) Statistics of first peak amplitude of target gene-knockdown cells (*n* >100 cells; data are acquired with three independent experiments and shown as mean ± SEM; **P* < 0.05, ***P* < 0.01, ****P* < 0.001 *versus* scrambled control).

**Supplementary Figure 4. Buffering Ca^2+^ oscillation inhibited spheroid formation of Hep-12 cells.** (**a**) Phase-contrast images of spheroids formed by Hep-12 cells under different conditions (scale bar: 100 μm). (**b**) Ca^2+^ buffers damp Ca^2+^ oscillation. Hep-12 cells were treated with 10 μM BAPTA-AM or 10 μM EGTA-AM. Statistics of oscillation frequency (*n* = 115 cells for BAPTA-AM treatment and 125 for EGTA-AM treatment; data are acquired with three independent experiments and shown as mean ± SEM; ****P* < 0.001 *versus* control).

**Supplementary Figure 5. Accelerated ER Ca^2+^ release after IP_3_R2 knockdown.** (**a**) Averaged responses of cytosolic and ER Ca^2+^ of IP_3_R2-knockdown Hep-12 cells to 40 μM ATP in Ca^2+^-free solution with ER Ca^2+^ uptake blocked by 4 μM Tg. (**b**) Statistics for the ER release kinetics (*n* = 96 cells in control, 111 for IP_3_R2 KD1, and 93 for IP_3_R2 KD2; data are acquired with three independent experiments and shown as mean ± SEM; ****P* < 0.001 *versus* control).

**Supplementary Movie 1**. Hep-12 cells exhibit robust Ca^2+^ oscillations in response to 40 μM ATP. Scale bar: 20 μm..

**Supplementary Movie 2**. Hep-11 cells exhibit non-oscillatory Ca^2+^ transients in response to 40 μM ATP. Scale bar: 20 μm.
