# Supplementary figures and images for: Central role of IP3R2-mediated Ca2+ oscillation in self-renewal of liver cancer stem cells elucidated by high-signal ER sensor
Source: Cell Death Dis. 2019 May 21;10(6):396. doi: 10.1038/s41419-019-1613-2 (PMC6529459; doi:10.1038/s41419-019-1613-2)

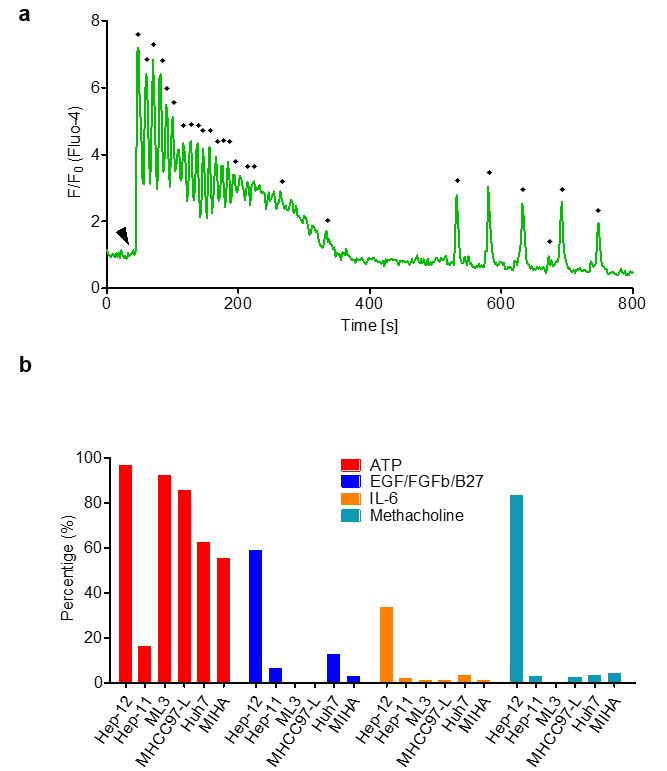

Supplement: Supplementary file 2 — Supplementary Figure 1 [file 41419_2019_1613_MOESM2_ESM.jpg]

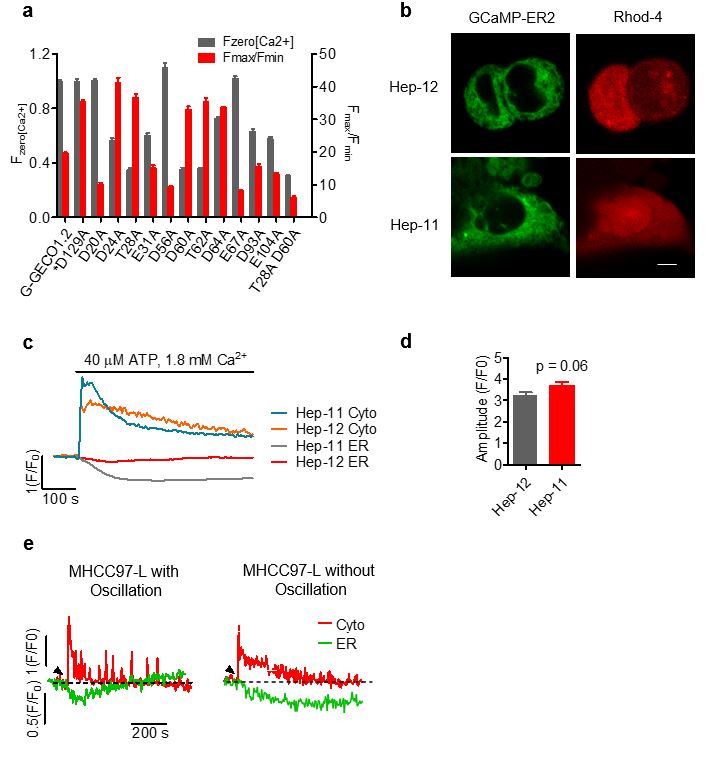

Supplement: Supplementary file 3 — Supplementary Figure 2 [file 41419_2019_1613_MOESM3_ESM.jpg]

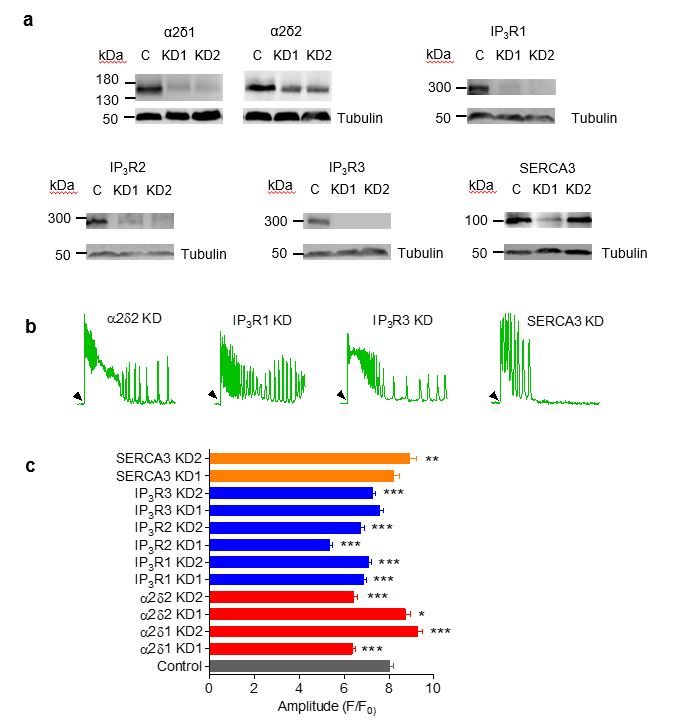

Supplement: Supplementary file 4 — Supplementary Figure 3 [file 41419_2019_1613_MOESM4_ESM.jpg]

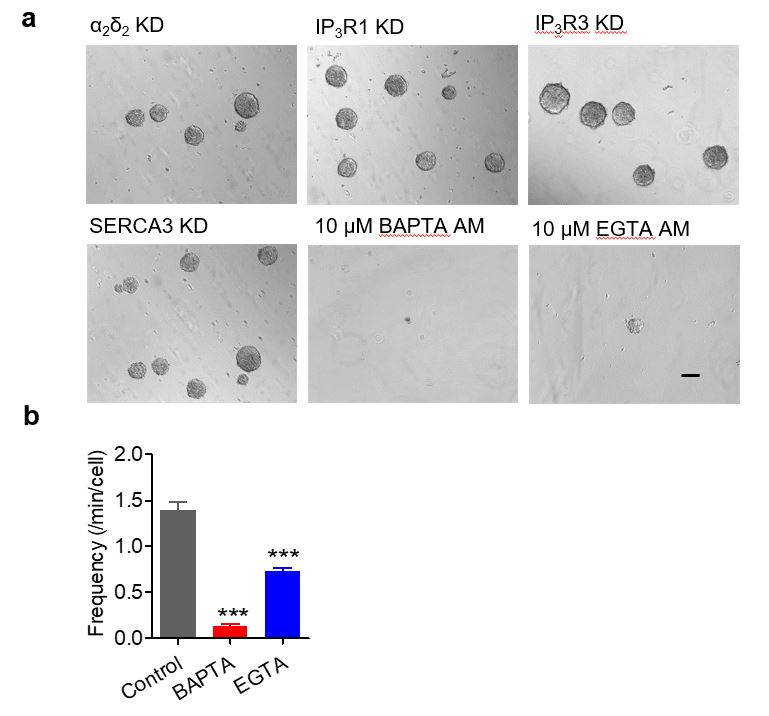

Supplement: Supplementary file 5 — Supplementary Figure 4 [file 41419_2019_1613_MOESM5_ESM.jpg]

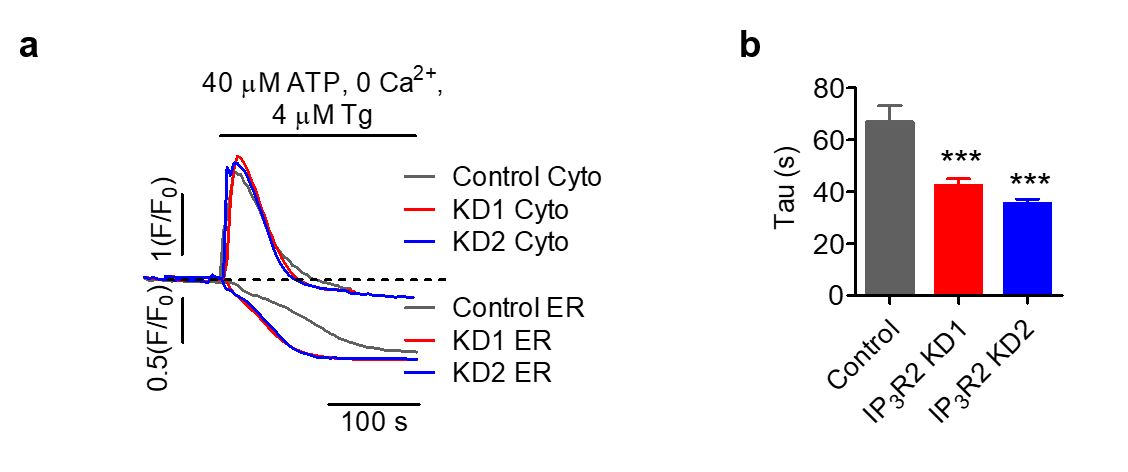

Supplement: Supplementary file 6 — Supplementary Figure 5 [file 41419_2019_1613_MOESM6_ESM.jpg]
